# Oral neomycin and bacitracin are effective in preventing surgical site infections in elective colorectal surgery: a multicentre, randomized, parallel, single-blinded trial (COLORAL-1)

**DOI:** 10.1007/s13304-021-01112-5

**Published:** 2021-06-20

**Authors:** Alberto Arezzo, Massimiliano Mistrangelo, Marco Augusto Bonino, Paola Salusso, Edoardo Forcignanò, Nereo Vettoretto, Emanuele Botteri, Nicola Cillara, Roberto Ottonello, Valentina Testa, Francesco Giuseppe De Rosa, Silvia Corcione, Roberto Passera, Mario Morino

**Affiliations:** 1 Department of Surgical Sciences, University of Torino, Corso Dogliotti 14, 10126 Torino, Italy; 2 Department of Surgery, Geneva University Hospitals, Geneva, Switzerland; 3 General Surgery, Ospedale Montichiari, Brescia, Italy; 4 Department of General Surgery, Santissima Trinità Hospital, Cagliari, Italy; 5 Department of Medical Sciences, University of Torino, Torino, Italy

**Keywords:** Oral antibiotics, Surgical site infection, Colorectal surgery

## Abstract

Several regimens of oral and intravenous antibiotics (OIVA) have been proposed with contradicting results, and the role of mechanical bowel preparation (MBP) is still controversial. This study aims to assess the effectiveness of oral antibiotic prophylaxis in preventing Surgical Site Infections (SSI) in elective colorectal surgery. In a multicentre trial, we randomized patients undergoing elective colorectal resection surgery, comparing the effectiveness of OIVA versus intravenous antibiotics (IVA) regimens to prevent SSI as the primary outcome (NCT04438655). In addition to intravenous Amoxicillin/Clavulanic, patients in the OIVA group received Oral Neomycin and Bacitracin 24 h before surgery. MBP was administered according to local habits which were not changed for the study. The trial was terminated during the COVID-19 pandemic, as many centers failed to participate as well as the pandemic changed the rules for engaging patients. Two-hundred and four patients were enrolled (100 in the OIVA and 104 in the IVA group); 3 SSIs (3.4%) were registered in the OIVA and 14 (14.4%) in the IVA group (*p* = 0.010). No difference was observed in terms of anastomotic leak. Multivariable analysis indicated that OIVA reduced the rate of SSI (OR 0.21 / 95% CI 0.06–0.78 / *p* = 0.019), while BMI is a risk factor of SSI (OR 1.15 / 95% CI 1.01–1.30 *p* = 0.039). Subgroup analysis indicated that 0/22 patients who underwent OIVA/MBP + vs 13/77 IVA/MBP- experienced an SSI (*p* = 0.037). The early termination of the study prevents any conclusion regarding the interpretation of the data. Nonetheless, Oral Neomycin/Bacitracin and intravenous beta-lactam/beta-lactamases inhibitors seem to reduce SSI after colorectal resections, although not affecting the anastomotic leak in this trial. The role of MBP requires more investigation.

## Introduction

Elective colorectal surgery is considered a clean-contaminated procedure, with a surgical site infection (SSI) rate of about 10% or above [1–3]. For many years, the role of Mechanical Bowel Preparation (MBP) has been universally recognized as an effective measure to reduce the colorectal bacterial load and consequently SSI rate, mostly in European Countries [4]. However, in the early 1970s, Nichols et al. showed a further SSI risk reduction in colorectal surgery if oral non-absorbable antibiotics were added to MBP [5], and for the next 30 years, this was suggested as the standard of care prior to elective colorectal surgery, especially in the US. More recently, the role of MBP has been questioned, being held by some to be responsible for an increased incidence of anastomotic dehiscence, somehow related to both bacterial translocation and electrolyte imbalances [6].

Not surprisingly, the assessment of the exact regimen of preoperative preparation for colorectal surgery is on one side still controversial. On the other side, national surveys show that adherence to the guidelines on prophylaxis is extremely poor both in terms of timing and duration of prophylaxis compared to what is recommended [7]. As a result, already in 2014, the Cochrane review on antimicrobial prophylaxis in colorectal surgery [8] identified 68 different therapeutic regimens in 260 different published trials, which substantially prevents any certain conclusion about the preferability of one therapeutic regimen over the others. As a consequence, at least in Italy, the adoption of oral antibiotic prophylaxis to reduce SSIs is still not common, and where it is adopted, it is performed with a variety of schemes.

We, therefore, conceived this study on a national basis to verify if oral antibiotic prophylaxis in addition to intravenous (iv) short-course antibiotic prophylaxis was associated with a reduction of SSI in elective colorectal surgery in our national reality.

## Materials and methods

The COLORAL-1 trial was a national multicentre, single-blinded, parallel-group, individually randomized superiority trial comparing preoperative oral and iv antibiotics prophylaxis (OIVA) with iv only antibiotics prophylaxis (IVA) in patients undergoing elective colorectal surgery. Initially conceived to enrol participants from at least 20 national centers, the trial was stopped after an interim analysis, at which stage 4 Italian hospitals belonging to the Italian Society for Endoscopic Surgery (SICE) were participating: 2 University hospitals (Torino University Hospital and Cagliari University Hospital) and two community hospitals (Montichiari Hospital Brescia and Santissima Trinità Hospital Cagliari). All participating hospitals are government-funded and provide care to all patients within their catchment area. The research plan was approved by the local Ethics Committee of the University of Turin (Prot. 0045543). The research plan was further approved by each participating centre's institutional review board. The trial was registered with ClinicalTrials.gov (NCT: 04438655).

### Participants: inclusion criteria

Consecutive patients who were scheduled for colorectal resection in participating centers for any indication (cancer, chronic diverticulitis, inflammatory bowel disease), > 18 years old and in general health condition permitting general anesthesia (ASA, American Society for Anaesthesiology classification I–III) were eligible for inclusion and recruited. Open, laparoscopic, laparoscopic-assisted, or laparoscopic converted to open were all suitable techniques, as well as any mechanical bowel preparation as indicated by each centre. All patients fulfilling the above-mentioned criteria were informed about the study by the physician. After consent was given, central web-based data acquisition took place. Patients were randomized into the two groups and treated according to the study protocol. Patients unable or refusing to provide informed consent were treated according to current clinical practice.

### Exclusion criteria

Exclusion criteria were as follows: the need for emergency surgery; appendectomy; primarily urological/gynaecological or vascular procedure; diagnostic laparotomy/laparoscopy without intestinal resection; surgery involving multi-visceral surgery (e.g. pelvic exenteration); contraindication for MBP; allergy to used drugs; patients who refuse to participate in the study; patients with intra-abdominal sepsis before surgery (abscess); patients who received antibiotics for any reason within two weeks prior to surgery; patients who do not comply strictly with the assigned prophylaxis regimen; patients who cannot be followed at least four weeks after surgery.

### Data collection

Per each participant, we collected data regarding age, gender, antibiotics administered, procedure and outcome, the American Society of Anaesthesiologists (ASA) score, cardiorespiratory and metabolic co-morbidities (including chronic obstructive pulmonary disease (COPD), chronic kidney disease (CKD), peripheral vascular disease (PVD) and diabetes mellitus, history of previous abdominal surgery, preoperative administration of immunosuppressive/steroid therapy, preoperative chemo-radiotherapy, and preoperative albumin serum level (g/dL) (Appendix).

### Randomization and masking

Patient data were entered into a centralized web-based database, and blind randomization was done by means of an unchangeable number-generating software programme. It was stratified according to the centre for right colectomy, left colectomy, or rectal resection. Patients were randomly assigned in a 1:1 ratio to either OIVA or IVA. The study recruiters had no further role in the trail after the randomization process. Nursing staff, operating surgeons, and treating physicians were masked to the allocated treatment. Patients and data collectors were not masked to treatment allocation.

### Procedures

All patients received standard iv prophylaxis at the time of induction of anesthesia, redosing with prolonged surgery: Amoxicillin/Clavulanic acid 2000/200 mg or, in the event of an allergy to Penicillin, Clindamycin 600 mg + Gentamycin 2 mg/kg. Dose adjustment was necessary for the presence of Creatinine Clearance, respectively < 30 ml/min and < 60 ml/min in accordance with pharmacologic recommendations. In addition, patients allocated to OIVA were instructed by the study recruiter to ingest Neomycin 25,000 UI and Bacitracin 2500 UI 24, 16 and 8 h before induction of anesthesia. The three pills were forwarded to the patient in due time in a package prepared by the hospital. Compliance was monitored by the caregiver. In the case of sepsis, iv antibiotics were continued according to the clinical indications.

The receipt of the allocated intervention was controlled by a nurse asking the patients on the morning of the surgery, whether they had acted as instructed by the allocation. This information was also concealed from treating physicians and surgeons, data collectors, and data analysts until the primary and secondary outcomes were analyzed. Patients were treated according to local protocols which were not changed for the study. Perioperative care followed the Enhanced Recovery After Surgery (ERAS) criteria [9], except for oral mechanical bowel preparation which was administered for some left-sided colonic, and all anterior resections with extra-peritoneal anastomosis. Furthermore, in all procedures ending with an extra-peritoneal anastomosis, a pelvic drain was left in place. Surgical skin preparation involved shaving the hair from the operation area on the morning of the operation day. Just before skin incision, the area was then washed with chlorhexidine and left to dry.

The surgical wounds were inspected daily by the surgeon responsible for the ward of the patients till discharge. When a deep incisional SSI was suspected, based on fever (> 38ºC), or localized pain, or tenderness, the wound was opened, removing some stitches. Wound cultures were performed only in case of doubt or persistent infection. No SSI was calculated in case of coexisting anastomotic leak. The patients were contacted 10, 20, and 30 days after the operation by a visit to the outpatient clinic. Patients were asked about any complications that had occurred after discharge, and clinical examination was carried out during visits to the outpatient clinic.

### Study endpoints

The primary endpoint was the incidence rate of surgical site infections (superficial or deep) at 30 days after index surgery. Patients who required further surgery for any reason different from SSI, including anastomotic leak, were excluded from the analysis. According to the CDC criteria [10] SSIs are classified as being either incisional or organ/space specific.

Superficial Incisional SSI includes purulent drainage from the external incision, organisms isolated from an aseptically obtained culture of fluid or tissue from the superficial incision, pain or tenderness, localized swelling, redness, or heat and external incision unless incision is culture-negative.

Deep Incisional SSI includes infections involving deep soft tissues (e.g., fascia and muscle layers) of the incision and purulent drainage from the deep incision but not from the organ/space component of the surgical site, or a deep incision spontaneously dehiscent associated with either fever (> 38 ºC), or localized pain, or tenderness unless the site is culture-negative, or an abscess or other evidence of infection involving the deep incision found on direct examination, during reoperation, or by histopathology or radiology examination.

*Secondary endpoints* were perioperative complications, anastomotic dehiscence, postoperative ileus, extra-abdominal complications, readmission, reoperation, length of hospital stay, mortality, and adverse effects of antibiotics (diarrhoea, Clostridium difficilis infection,) all checked at 30 days from operation. Anastomotic leak was suspected by clinical examination (fever, abdominal pain, and ileus) in combination with blood tests (leucocytosis, increased C-reactive protein levels) and confirmed by an abdominal CT scan and/or laparotomy. Abscesses in the proximity of the anastomosis were also considered an anastomotic leak. Complications are classified in accordance with Dindo–Clavien's classification [11].

### Follow-up

Patients were followed for at least 30 days after surgery. All secondary outcome measures were recorded if they occurred at any point from postoperative day 0 (day of surgery) to day 30. The follow-up of patients included a clinical evaluation 30 days after surgical intervention supported by blood analysis (WBC count and CRP) to completely exclude the presence of any infectious complication. The presence of fever and/or WBC count/CRP elevation was further investigated with radiological imaging and was considered an infectious postoperative complication (SSI or not).

### Sample size determination and statistical analysis plan

On the basis of personal and literature historical data, an SSI rate of 16.3% (95%CI 13.2–20.0) was expected in elective colorectal laparoscopic surgery when standard intravenous short-term antibiotic prophylaxis is administered. The SSI rate was 6.8% when intravenous short antibiotics prophylaxis was administered in association with oral non-absorbable antibiotics (95% CI 5.6–8.4). Statistical analysis showed that considering the closest limits of the two CI intervals (13.2 and 8.4%), with a *β*-error of 0.20 and a one-sided *α*-error of 0.05, 656 patients were needed per group.

An interim analysis was performed at the time the COVID-19 pandemic was declared. The interim analysis, carried out by an independent statistician and reviewed by the statistician involved in the trial, was performed to evaluate whether the power was still sufficient to continue the trial. The final decision to stop the trial was made by the principal investigator of the trial.

No surgeon-, hospital-, or country-specific comparisons were performed. The main analyses were performed on an intention‐to‐treat basis, without the exclusion of patients after randomization.

The data were analyzed using descriptive (absolute/relative frequencies for categorical variables and median/IQR Inter Quartile Range for continuous ones) and inferential (Fisher ‘s exact test and Mann–Whitney test, respectively) statistics as well as uni/multivariable binary logistic regression models.

Odds ratios (ORs) were estimated for the binary main outcome measure (occurrence of SSIs) by a series of logistic regression models; potential confounders for complication rates, identified on the literature, were age, gender, BMI, ASA classification, smoking history, diabetes, bowel preparation, a surgical procedure performed, diverting ileostomy, and conversion. In a subgroup analysis, we divided patients between those who underwent MBP and those who did not.

All the analyses were performed by R 4.0.2 (R Foundation for Statistical Computing, Vienna-A, http://www.R-project.org).

### Data collection and governance

Data were collected and stored online. Communication between the clients and the server where it was hosted the online platform was secured under TLS protocol with an encryption certificate SHA256 with RSA 2048 bits (e 65537). To maximize the data protection, the physical HD where the database was stored was encrypted too. Access to the online platform was possible only for the previous Login. Each physician was able to see and modify only the data of patients added from his/her centre. The online platform checked the validity and the correct format of the fields and generated an excel table to import to the statistic software.

## Results

**Fig. 1:**
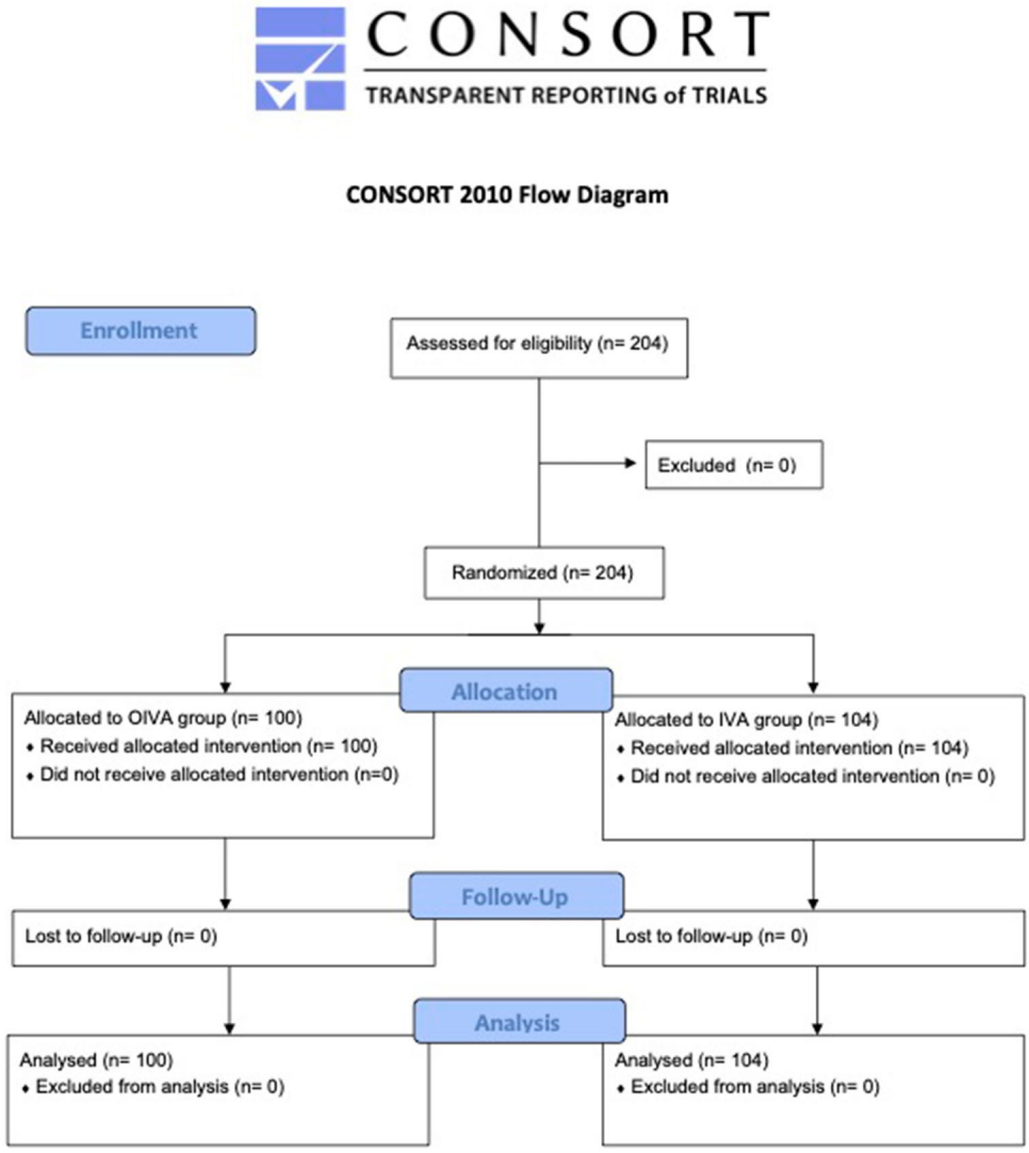
CONSORT diagram for the study

A total of 204 patients were enrolled from July 1st, 2019 to June 1st, 2020 (Fig. 1). The trial was stopped after the upheaval that the COVID-19 pandemic has brought to our country before others. This has significantly changed the priorities at this time, as well as altered the methods of enrolling and managing eligible patients for the study, both in ours and in other hospitals which were willing to take part in the trial. This led us to foresee an unreasonable duration of the study until the sample was completed, which would invalidate the scientific validity of the study itself. For the aforementioned reasons, it was considered mandatory to interrupt the study.

Administration of OIVA was according to the protocol in 100 patients (49%). Another 104 received IVA (51%). The median age of the whole cohort was 70 (IQR 25–95) years, and 109 of them were male (53.4%). The ASA score had a comparable distribution between the two arms: 61 OIVA subjects (61%) had an ASA I–II, while 69 patients (66.3%) had the same score in the IVA arm (*p* = 0.471). The median BMI was 24 (IQR 14.9–34.2), while 34 were active smokers (16.7%). The median preoperative albumin was 4.1 (IQR 2.8–5.1) g/dl, the median operative time was 160 min (IQR 35–401), and the median hospital stay was 7 days (3–70).

One hundred and two patients (50%) received either a right or left colectomy, 48 (48%) in the OIVA group and 54 (62%) in the IVA group. All of them did not receive preoperative bowel mechanical preparation.

55 (26.9%) patients underwent either a partial or a total mesorectal excision, 34 (34%) in the OIVA group and 27 (26%) in the IVA group. 50 (90%) of them received preoperative bowel mechanical preparation.

Baseline characteristics of patients included are shown in Table 1.

**Table 1:** Baseline characteristics of individuals

	OIVA (*n* = 100)	IVA (*n* = 104)
Gender ratio (F /M)	47 (47%)/53 (53%)	48 (46.2%) / 56 (53.8%)
BMI (kg/m^2^), median (IQR)	24.1 (16.4–34.2)	23.9 (14.9–33.4)
ASA fitness grade		
I (healthy)	7 (7%)	11 (10.6%)
II (mild systemic disease)	54 (54%)	58 (55.8%)
III (severe systemic disease)	39 (39%)	35 (33.7%)
Diabetes, *n* (%)	17 (17)	14 (13.5)
Preoperative albumin level (g/dl), median (IQR)	4.1 (3–5.1)	4.1 (2.8–5)
Active smoker, *n* (%)	19 (19)	15 (14.4)
Neoadjuvant therapy, *n* (%)	17 (17)	15 (14.4)
Mechanical bowel preparation, *n* (%)	26 (26)	24 (23.1)
Surgical intervention, *n* (%)		
Right colectomy	27 (27)	30 (28.8)
Extended Right colectomy	4 (4)	2 (1.9)
Ileo-colic resection	2 (2)	4 (3.8)
Transverse colectomy	5 (5)	8 (7.7)
Left colectomy	21 (21)	24 (23.1)
Subtotal colectomy	5 (5)	3 (2.9)
Rectal anterior resection (PME-TME-TaTME)	32 (32)	23 (22.1)
Hartmann procedure	2 (2%)	3 (2.9%)
Other	2 (2%)	7 (6.7%)
Type of surgery, *n* (%)		
Laparoscopic	80 (80)	82 (78.8)
Open	11 (11)	11 (10.6)
Diverting ileostomy	12 (12)	9 (8.7)
Conversion	9 (9)	11 (10.6)

Values in parentheses are percentages for categorical variables and IQR for continuous ones

*PME* = partial mesorectal excision, *TME* = total mesorectal excision, *TaTME* = trans-anal total mesorectal excision, *OIVA* = oral and in vein antibiotics, *IVA* = in vein antibiotics

### Clinical outcomes

Seventeen SSIs globally occurred (9.1%), 3 (3.4%) in the OIVA group and 14 (14.4%) in the IVA one (*p* = 0.010).

An anastomotic leak was recorded in 10 patients (10%) in the OIVA group and 8 (7.7%) in the IVA group (*p* = 0.805). Five patients in the OIVA group (5%) with anastomotic leak required reoperation, and the remaining 3 underwent drainage either percutaneously or via the rectum. In the IVA group, 8 patients required reoperation (7.6%) and 2 were drained percutaneously or via the rectum (Table 2). Therefore, no difference was observed in the rate of second surgery (*p* = 0.329).

**Table 2:** Results of primary and secondary outcomes

	OIVA (*n* = 100)	IVA (*n* = 104)	*p*
Surgical‐site infection	3 (3.4%)	14 (14.4%)	0.010
Anastomotic leak	10 (10%)	8 (7.7%)	0.805
Required intervention for anastomotic leak			
Reoperation	5	8	0.329
Conservative treatment	3	2	
Extra abdominal complications	18 (18%)	19 (18.3%)	0.856
Cardiac	4 (22.2%)	2 (10%)	
Pulmonary	3 (16.7%)	5 (26.3%)	
Urinary tract	2 (10.5%)	0 (0%)	
Anaemic	3 (16.7%)	4 (21.1%)	
Other	5 (27.7%)	8 (42.1%)	
Re-admission	3 (3%)	3 (2.9%)	1.000
30‐day mortality	4 (4%)	1 (1.0%)	0.205

Values in parentheses are percentages for categorical variables and IQR for continuous ones

*OIVA* oral and in vein antibiotics, *IVA* in vein antibiotics

Eighteen patients (18%) in the OIVA group had one or more complications (infectious and non-infectious) compared with 19 (18.3%) in the IVA group (*p* = 0.856). No difference was recorded between groups for incidence of pneumonia, urinary tract infection, or other infections. No infections with multidrug‐resistant bacteria or Clostridium difficilis were reported.

The 30‐day mortality rate did not differ between the two arms: four deaths (4%) occurred in the OIVA group and one (1.0%) in the IVA group (*p* = 0.205). No differences were found in the median time to first oral intake, first bowel movement, median hospital stay, readmission, and reoperation within 30 days (Table 2).

### Univariable and multivariable binary logistic regression models

The occurrence of SSIs (binary dependent variable) has been investigated by a series of logistic regression models. In the univariable models, the two most important predictors (independent variables) were OIVA administration (OR 0.21 / 95% CI 0.06–0.75 / *p* = 0.016) and BMI (modelled as a continuous covariate, OR 1.15 / 95% CI 1.01–1.30 *p* = 0.039).

The multivariable model sharply confirmed these findings; OIVA (OR 0.21 / 95% CI 0.06–0.78 / *p* = 0.019) and BMI (OR 1.18 / 95% CI 1.02–1.36 *p* = 0.027) were the critical risk factors for SSIs occurrence (Table 3).

**Table 3:** Univariable and multivariable binary logistic regression models for the risk of SSI

Univariable models			
	OR	CI 95%	*p*
OIVA vs IVA	0.21	0.06–0.75	0.016
MBP vs No MBP	0.19	0.03–1.51	0.117
BMI	1.15	1.01–1.30	0.039

Multivariable model #1			
	OR	CI 95%	*p*
OIVA vs IVA	0.21	0.06–0.78	0.019
MBP vs No MBP	0.13	0.03–1.59	0.129

Multivariable model #2			
	OR	CI 95%	*p*
OIVA vs IVA	0.18	0.05–0.67	0.010
BMI	1.18	1.02–1.36	0.022

*OIVA* oral and in vein antibiotics, *IVA* in vein antibiotics, *MBP* mechanical bowel preparation, *SSI* surgical site infection, *BMI* body mass index, *OR* odds ratio, *CI* confidence interval

Afterwards we restricted the analysis to the cohort of 154 patients who did not undergo MBP. Again, in the univariable models, the two most important predictors (independent variables) were OIVA administration (OR 0.23 / 95% CI 0.06–0.86 / *p* = 0.029) and BMI (OR 1.19 / 95% CI 1.03–1.38 / *p* = 0.017). Similarly, the multivariable model sharply confirmed these findings OIVA administration (OR 0.24 / 95% CI 0.06–0.90 / *p* = 0.034) and BMI (OR 1.20 / 95% CI 1.03–1.39 / *p* = 0.021) confirmed to be the critical risk factors for SSIs occurrence.

### Subgroup analysis

Excluding patients in which anastomotic dehiscence was observed, we performed a subgroup analysis considering patients who underwent MBP (22 patients in the OIVA group, 20 patients in the IVA group) and those who did not (66 patients in the OIVA patients, 77 patients in the IVA group) (Table 4). Of 17 SSIs observed, 16 (94.2%) occurred in patients who did not undergo MBP and only one (5.8%) in a patient who performed MBP (Table 4). Of the 16 patients who experienced an SSI and did not undergo MBP, 3 were in the OIVA group (3/66) compared to 13 in the IVA group (13/77) (*p* = 0.031). The only patient who experienced an SSI among those who received MBP was recorded in the IVA group. No patient who received MBP in the OIVA group registered an SSI.

**Table 4:** Incidence of SSI divided per antibiotic and mechanical bowel preparation

	IVA, no MBP	OIVA, no MBP	IVA, MBP	OIVA, MBP	Total
No SSI (%)	64 (38.1)	63 (37.5)	19 (11.3)	22 (13.1)	168 (100)
SSI (%)	13 (76.5)	3 (17.6)	1 (5.9)	0 (0)	17 (100)
Total (%)	77 (41.6)	66 (35.7)	20 (10.8)	22 (11.9)	185 (100)

Values in parentheses are percentages for categorical variables and IQR for continuous ones

*OIVA* oral and in vein antibiotics, *IVA* in vein antibiotics, *MBP* mechanical bowel preparation, *SSI* surgical site infection

We compared each combination of antibiotic regimen and MBP with each other (Table 5). Patients who underwent both oral and in-vein antibiotics combined with MBP (OIVA/MBP +) observed less incidence of SSI than patients who underwent only in-vein antibiotics and did not perform any MBP (IVA/MBP-): 0/22 versus 13/77 (*p* = 0.037).

**Table 5:** Subgroup analyses varying OIVA and IVA with or without MBP

Subgroup 1	Subgroup 2
OIVA/MBP + SSI/Tot (%)	IVA/MBP + SSI/Tot (%)
0/22 (0%)	1/20 (5%)
OIVA/MBP- SSI/Tot (%)	IVA/MBP− SSI/Tot (%)
3/66 (4.5%)	13/77 (16.9%)
OIVA/MBP + SSI/Tot (%)	OIVA/MBP− SSI/Tot (%)
0/22 (0%)	3/66 (4.5%)
OIVA/MBP + SSI/Tot (%)	IVA/MBP− SSI/Tot (%)
0/22 (0%)	13/77 (16.9%)
IVA/MBP + SSI/Tot (%)	IVA/MBP− SSI/Tot (%)
1/20 (5%)	13/77 (16.9%)
IVA/MBP + SSI/Tot (%)	OIVA/MBP− SSI/Tot (%)
1/20 (5%)	3/66 (4.5%)

Values in parentheses are percentages for categorical variables

*OIVA* oral and in vein antibiotics, *IVA* in vein antibiotics, *MBP* mechanical bowel preparation, *SSI* surgical site infection

## Discussion

Historical arguments in favour of the use of oral antibiotic prophylaxis in association with the mechanical preparation are undoubtedly the reduction of endogenous bacteria, including anaerobes, the decrease of SSIs, and the reduction of the post-surgical ileum, albeit with the same incidence of *Clostridium difficilis* infections [12, 13]. Arguments against the use of oral antibiotic prophylaxis associated with mechanical intestinal preparation are the heterogeneity of the proposed protocols that did not make clear the results. In addition to an undoubted greater difficulty in organizing the preoperative workflow, there is a resistance from patients to the intake, especially of intestinal preparation. On the other hand, the need for a randomized-controlled trial (RCT) on the subject is well known, as studies on the use of the oral antibiotic exclusively contradictory [14–16]. This need was only partially met with recent studies proposed by Espin et al. [17]. Finally, more than running the risk of generating antibiotic resistance, it is well known the impact that antibiotics have on the gut microbiome that lasts well beyond the surgical intervention [18, 19].

The present study shows that the use of oral Neomycin in addiction to bacitracin in triple administration for the 24 h preceding the intervention, and in association with amoxicillin and clavulanic acid in-vein at the induction of anesthesia and for 24 h after the intervention, significantly reduces the risk of SSI compared to only antibiotic prophylaxis in the vein. Risk factors for SSI are manifold [20], and the methods to decrease the risk are, therefore, different. Antibiotic prophylaxis is known to play a fundamental role. However, in Italy, the adoption of oral antibiotic prophylaxis to reduce SSIs is not common. We, therefore, wanted to propose this study to verify if different factors contributed to a different result than expected. Here, antibiotic prophylaxis is performed more frequently with beta-lactamase inhibitors. However, recently, the American Society for Colo-Rectal Surgery (ASCRS) guidelines suggest the use of 1-day prophylaxis with second-generation cephalosporins [21, 22]. However, the greatest difficulties are undoubtedly in the choice of the oral antibiotic, given the now scarce availability of erythromycin, which seemed to be the first choice. Our choice, therefore, fell on the molecule most used in Italy for oral antibiotic prophylaxis and therapy, namely Neomycin, which in Italy is offered by the pharmaceutical handbook exclusively in association with bacitracin. Our results show that this combination of three antibiotics, two oral and one iv, is at least as effective as other regimens proposed in the reduction of SSI after elective colorectal surgery.

The role of antibiotic prophylaxis itself in association with MBP remains uncertain. MBP is no longer recommended as being considered a non-determining factor in the risk of anastomosis dehiscence if not actually counterproductive [23], remaining indicated only in extra-peritoneal rectal anastomoses [24]. In the present study, the operators were left free to perform the MBP or not according to the protocol in place at the respective institutions, without changes. This led to the enrolment of patients undergoing elective colonic resection in all but 7 cases without MBP, while all patients undergoing rectal resection were all mechanically prepared. We observed that the incidence of SSI is higher in the IVA group with a statistically significant difference and a clear prevalence of events (SSI) in the group that did not perform MBP. These data confirm the protective role of the oral antibiotic, in addiction to in-vein antibiotic prophylaxis on the incidence of SSI regardless of MBP. The role of MBP with respect to this outcome remains; however, uncertain and data of our study suggest a probable protective role of MBP against SSIs, more evident in association with the oral antibiotic, possibly due to a synergic action. Nevertheless, any statistical deduction would be incorrect, since the SSI event is known to be determined by multiple and often confounding factors. Furthermore, the number of patients undergoing preparation is too meagre, and much larger sample size is needed to clarify this point. Nor should it be forgotten that the choice of whether to prepare patients or not was left to individual centers according to local habits and that, in our study, this corresponded in the majority of cases with limiting MBP to patients undergoing anterior rectal resections with sub-peritoneal anastomoses.

At the same time, our study showed that the present regimen of oral and in-vein antibiotic prophylaxis did not influence the risk of anastomotic leak. Here, in recent years, the role of the microbiome has gradually established itself. Today, it is well recognized that the diversity and metabolic interactions of this microbial community greatly influence the development of infection and disease [25]. The possible lost in balance in the microbiome, which facilitates the predominance of potentially pathogenic microorganisms in the bowel, is considered favouring infectious complications [26]. It is known that the vast majority of SSIs following colorectal surgery are caused by endogenous bacteria [27]. On this, a fundamental role is played by both the disruption of the mucosal barrier integrity during surgery and the alteration of the composition of the microbiome due to the postoperative ileus. On the other side, it is likely that the microbiome may play a fundamental role also in the pathogenesis of an anastomotic leak, but how the mechanism takes place is still a matter of research. In fact, OIVA results in selective decontamination of the gut, being based on non‐absorbable antibiotics, this way minimizing the impact of endogenous flora.

There are several limitations to this study. The analysis of the results of this study is undoubtedly burdened by the decision to interrupt the same for the contingent state of emergency for pandemic linked to COVID-19. In fact, first the difficulty in finding active centers even though their participation had been approved, then the occurrence of a sudden interruption in the performance of elective surgery including also oncological cases, and the persistence of a partial recovery only, made the steering committee of the study believe that it was correct to stop the sweat. On this, it has certainly weighed on one hand the uncertainty of the continuation of the situation of the pandemic, on the other hand, the change in the rules for engaging patients, on which the calculation of the sample size of the present study was based on. Specifically, the fact of not allowing anyone, except the patient himself, to enter the hospital for the duration of the hospitalization, but, above all, the marked increase in the rigour in the use of safeguards to limit the risk of transmission of infection, from hand washing to the use of masks, certainly played a role in the occurrence of events such as SSIs. Furthermore, the termination of the study did not allow to reach a sufficient number of participants for credible subgroup analysis.

In conclusion, the early termination of the study due to pandemic prevents us from reaching firm conclusions regarding the interpretation of the data. Nonetheless, we were able to observe that even the association of oral Neomycin and iv beta-lactams/beta-lactamase inhibitors seems effective in reducing infectious complications after elective colorectal resection without significantly affecting the rate of anastomotic leak, refuelling the old debate of whether orally administered antibiotics are useful in addition to iv prophylaxis.

## Data Availability

The datasets analyzed during the current study are available from the corresponding author on reasonable request.

## Declarations

### Research involving human and/or animal participants

All the investigators conducted the study following the principles of the Declaration of Helsinki of 1975 (as revised in 2008).
